# Ag_2_(0) dimers within a thioether-functionalized MOF catalyze the CO_2_ to CH_4_ hydrogenation reaction

**DOI:** 10.1038/s41598-023-37600-4

**Published:** 2023-06-27

**Authors:** Yongkun Zheng, Nuria Martín, Mercedes Boronat, Jesús Ferrando-Soria, Marta Mon, Donatella Armentano, Emilio Pardo, Antonio Leyva-Pérez

**Affiliations:** 1 Instituto de Tecnología Química (UPV–CSIC), Universidad Politècnica de València–Consejo Superior de Investigaciones Científicas, Avda. de los Naranjos s/n, 46022 Valencia, Spain; 2 Departamento de Química Inorgánica, Instituto de Ciencia Molecular (ICMol), Universidad de Valencia, Catedrático José Beltrán Martínez, 2, 46980 Paterna, Valencia Spain; 3 Dipartimento di Chimica e Tecnologie Chimiche (CTC), Università della Calabria, 87036 Rende, Cosenza Italy

**Keywords:** Heterogeneous catalysis, Nanoscale materials, Energy

## Abstract

Ultrasmall silver clusters in reduced state are difficult to synthesize since silver atoms tend to rapidly aggregate into bigger entities. Here, we show that dimers of reduced silver (Ag_2_) are formed within the framework of a metal–organic framework provided with thioether arms in their walls (methioMOF), after reduction with NaBH_4_ of the corresponding Ag^+^-methioMOF precursor. The resulting Ag_2_-methioMOF catalyzes the methanation reaction of carbon dioxide (CO_2_ to CH_4_ hydrogenation reaction) under mild reaction conditions (1 atm CO_2_, 4 atm H_2_, 140 °C), with production rates much higher than Ag on alumina and even comparable to the state-of-the-art Ru on alumina catalyst (Ru–Al_2_O_3_) under these reaction conditions, according to literature results.

## Introduction

Ligand-free sub-nanometric metal clusters are metastable chemical species where most of the atoms are sited at the surface of the particle, available to interact with external molecules^1,2^. This topology, combined with electronic and cooperative effects, makes metal clusters particularly useful in catalysis, since all metal atoms can participate during the catalytic chemical events^3,4^. From an economy and efficiency point of view, the smaller the cluster is the better the catalytic production per metal atom can be, the limit being on single metal atoms (single atom catalysts or SACs)^5^. However, the latter (SACs) lack potential metal-to-metal cooperation processes, which can be of undoubtable importance during the catalytic reaction. Following this rationale, metal dimers (two atoms) bring the best of both worlds for catalysis: optimized metal atom efficiency and metal cooperativity.

Coinage metal atoms are particularly suitable to form ultrasmall metal clusters, including dimers, since their electron-rich, loosely bound delocalized valence shells allow to bind few metal atoms in stable entities, without extensive aggregation under selected reaction conditions^6^. Not in vain, gold^7^, palladium^8^ and platinum^9^ clusters are commonly found in catalysis. However, silver is an exception here, since the extraordinary tendency of silver atoms to reduce (even with just light) and aggregate in nano- and micro-particles hampers the formation of ultrasmall metal clusters^10,11^. In particular, catalytic ligand-free silver dimers in reduced state (Ag_2_) have been obtained with electrochemistry techniques^12^, under size-selected synchrotron conditions^13–15^ or by supporting them in porous solids^16^.

During the last two decades, a type of porous materials, so-called metal–organic frameworks (MOFs), have been intensely studied given their excellent performances in many important fields ^17,18^, which mainly arise from their unique host–guest chemistry^19,20^. For instance, MOFs have been shown to be very effective for the encapsulation of different ultrasmall metal clusters^21^ and even for their in-situ step-by-step preparation within the functional channels of the MOF^4,22^, which acts as an efficient chemical nanoreactor. In this context, we have recently reported that the use of a highly robust and crystalline MOF, as a chemical nanoreactor, allowed to synthesise and stabilize Ag_1_ and Ag_2_ within the microporous networks, in gram amounts^23^. Our synthetic approach required the use of extensive solid-to-solid post-synthetic steps, including at least three metal cation exchanges before the final reduction of the Ag^+^–MOF precursor, which could be achieved given the high robustness and crystallinity of the selected MOF^23^.

In the present work, a previously reported MOF, with formula {Ca^II^Cu^II^_6_[(*S,S*)-methox]_3_(OH)_2_(H_2_O)}·16H_2_O (**1**)^24^ [where methox is bis[(*S*)-methionine]oxalyl diamide], whose channels are densely decorated with thioether arms, is efficiently used as chemical reactor to prepare Ag_2_ nanoclusters within its functional channels. Thus, the functional pore environment of **1**, considering the high affinity of sulfur towards silver atoms, enables the incorporation and chemical reduction of Ag^+^ in the MOF’s structure without additional steps, to generate the targeted **Ag**^**0**^**@1** with precise atomicity and in gram-scale^25^. This new solid material catalyzes very efficiently the hydrogenation of carbon dioxide (CO_2_) to methane (CH_4_) under mild reaction conditions (1 atm CO_2_, 4 atm H_2_, 140 °C), with productivity rates comparable to an industrial catalyst under these reaction conditions, according to the literature^26,27^. **Ag**^**0**^**@1** is relatively stable under reaction conditions and can be recovered and reused at least three times without severe depletion of the catalytic activity. These results bring ultrasmall Ag^0^ species as a new catalytic tool for hydrogenation reactions, moreover considering the reluctance of the CO_2_ molecule towards the hydrogenation reaction. Besides, the synthesis of this material, which consists in the formation of the MOF from highly available amino acids and calcium and copper salts^24^, and the incorporation of Ag and its subsequent reduction, proceeds with a very high atomic economy, since the only reagent that does not incorporates directly in the material is the final reducing agent, and all steps occur at room temperature. Therefore, the MOF synthesis is not neither energetically intensive nor material costly, so with a low carbon footprint, amply compensated in terms of global warming effects during the catalytic carbon dioxide hydrogenation reaction.

## Results and discussion

### Synthesis and characterization of 1, Ag^+^@1 and Ag^0^@1

**Figure 1:**
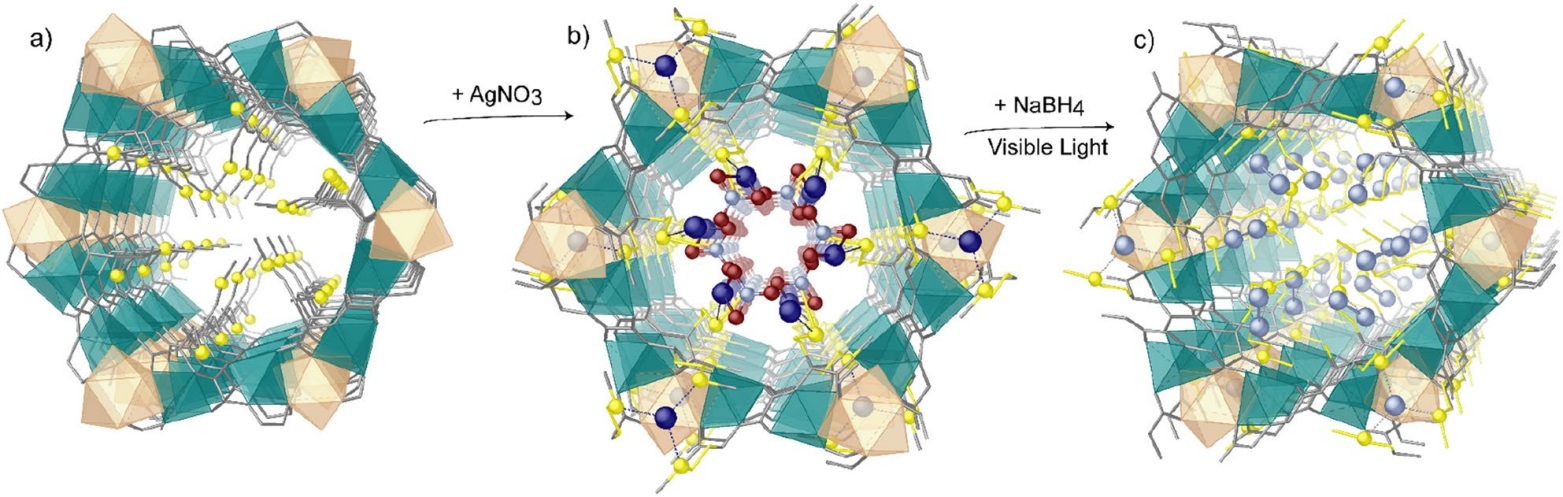
The schematic (based on the three real crystal structures) showing the two-steps protocol starting from **1** (**c**), soaked in AgNO_3_ aqueous solution to give **Ag**^**+**^**@1** (**b**) before preparation of **Ag**^**0**^**@1** (**c**). Color scheme: sulfur, yellow spheres; calcium, pastel orange polyhedra; copper, dark cyan polyhedra, silver(I), blue spheres, silver(0) sky blue spheres, oxygen and nitrogen from nitrate anions, red and violet spheres, atoms from the ligand, gray sticks.

**Figure 2:**
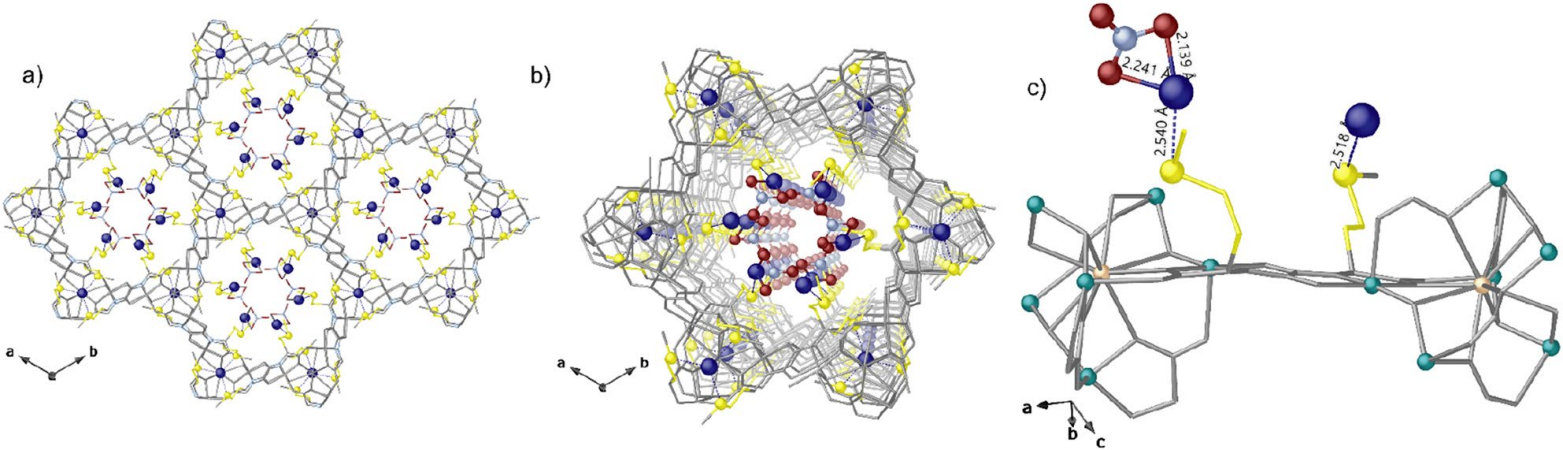
Crystal structure of **Ag**^**+**^**@1:** views of a fragment (**a**) and one single channel (**b**) of **Ag**^**+**^**@1** along the *c* axis; details of Ag^+^–S binding sites (**c**). Yellow and blue spheres represent S and Ag atoms whereas all the porous network is depicted as gray sticks. Sky blue and red spheres represent N and O atoms of nitrate anions. Blues dashed lines represent the Ag···S interactions. In (**c**) copper and calcium atoms from the network are represented by cyan and pastel orange spheres, respectively.

**Figure 3:**
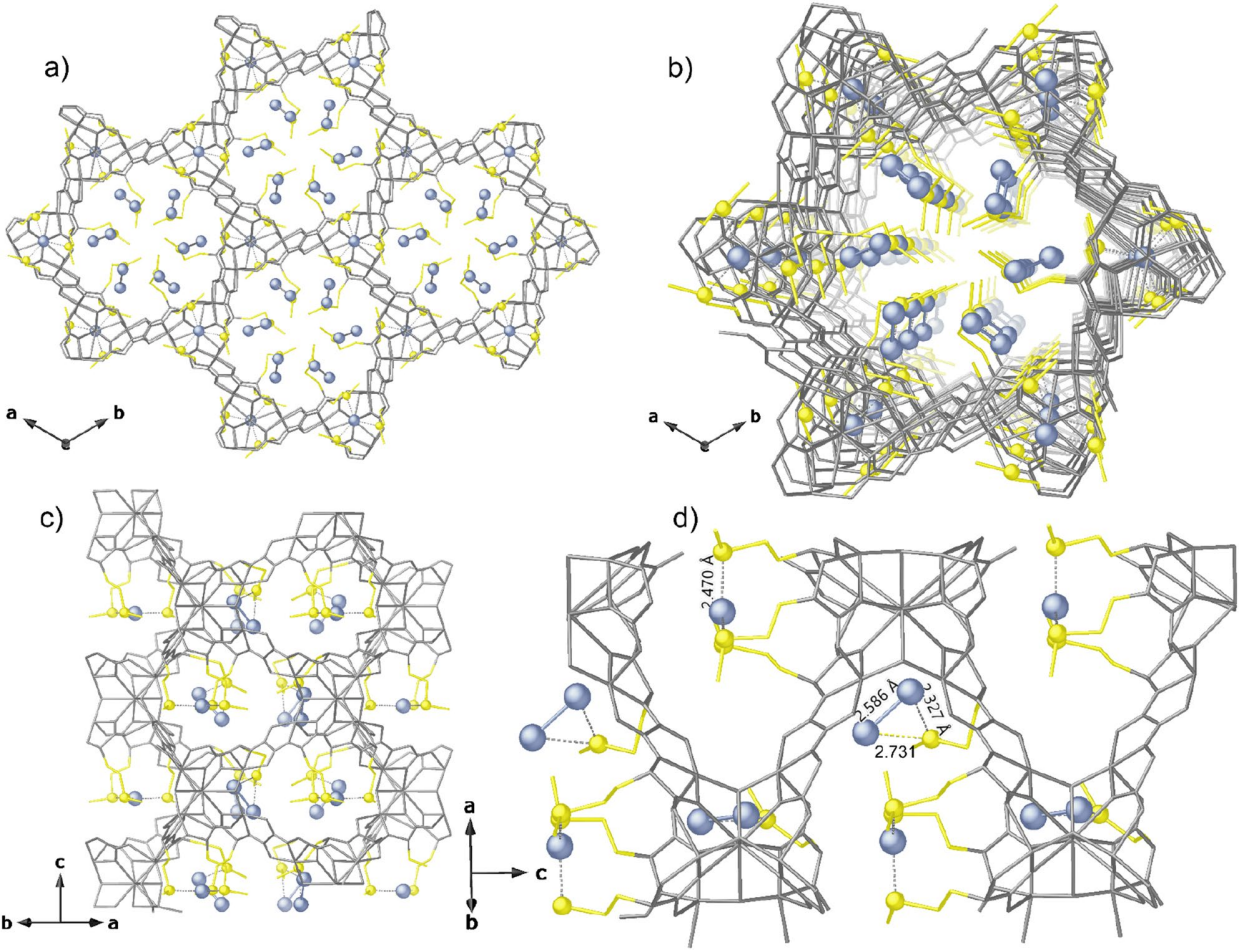
Crystal Structure of **Ag**^**0**^**@1**. Views of a fragment (**a**) and one single channel (**b**) along the c and [111] direction (**c**); Details of crystal structure and binding sites for Ag^0^_1_ and Ag^0^_2_ (**d**). Yellow and light blue spheres represent S and Ag^0^ atoms, respecively whereas all the porous network is depicted as gray sticks except for methionine arms represented as yellow sticks. Light blue dashed lines represent the Ag···S interactions.

MOF **1** was prepared as previously reported. The MOF-driven preparation of Ag^0^_1_ single atoms and Ag_2_ clusters takes place in two consecutive steps (Fig. 1). Firstly, the highly robust water-stable MOF **1**, featuring channels decorated with sulfur-containing chains capable to retain silver(I) cations, was soaked in a saturated AgNO_3_ aqueous solution to give an intermediate material with formula (AgNO_3_)_3_@{Ca^II^Cu^II^_6_[(S,S)–methox]_3_(OH)_2_(H_2_O)} ^.^ 13H_2_O (**Ag**^**+**^**@1**) (Fig. 2). Then, Ag^0^_1_ single atoms, and the Ag_2_ dimers could be obtained by reducing **Ag**^**+**^**@1** with NaBH_4_, under visible light, yielding the final material (Ag^0^_1_)(Ag^0^_2_)@{Ca^II^Cu^II^_6_[(S,S)-methox]_3_(OH)_2_(H_2_O)}^·^14H_2_O (**Ag**^**0**^**@1**) (Fig. 3).

Indeed, functional thio–alkyl chains decorating the pores of MOF **1** played a dual key role by retaining silver(I) cations within the MOF channels and also by limiting the number of silver(I) and allowing their homogeneous distribution along the channels (see structural section) cations inserted and allowing the ulterior formation of ultrasmall silver single atoms and clusters after reduction.

Regarding the synthetic protocol, single crystals of the MOF **1**, suitable for single crystal X-ray diffraction (SCXRD), could be obtained by slow diffusion techniques (see Experimental Section for complete description), as previously reported^24,28^. Then, given the high crystallinity and robustness of **1**, crystals of the MOF were used to obtain crystals of **Ag**^**+**^**@1** and **Ag**^**0**^**@1** after a soaking and reduction process, respectively, whose structures could be solved using synchrotron radiation. The obtention of the crystal structures of **Ag**^**+**^**@1** and **Ag**^**0**^**@1** allowed unique insights about the clusters nature, as described in the crystal structure section. Alternatively, a multi-gram protocol was also developed to obtain large quantities of polycrystalline samples of **1**, **Ag**^**+**^**@1** and **Ag**^**0**^**@1** (see Experimental Section) which were used for catalytic experiments.

The crystal structures of both **Ag**^**+**^**@1** and **Ag**^**0**^**@1** are isomorphic to **1**. They both crystallise in the *P*6_3_ chiral space group of the hexagonal system confirming the robustness of the 3D porous network of **1**, after AgNO_3_ capture and even after Ag^+^ reduction. In the crystal structure of **Ag**^**+**^**@1**, Ag^+^ metal ions together with NO_3_^-^ anions are trapped in the hexagonal nanopores of **1**, being recognized by the thioether arms of the methionine residues. Thanks to the robustness of the network, detailed analysis of synchrotron X-ray data (at T = 45 K) gave **Ag**^**0**^**@1** crystal structure allowing also to give new insights on the structural parameters and binding motifs of Ag_2_ clusters in MOF structure.

Crystal stucture of **Ag**^**+**^**@1** unambigueusly shows silver(I) cations captured and hosted in hydrophilic hexagonal pores [virtual diameter of 0.8 nm] open-framework structure of **1** (Figs. 1a,b, 2) and smaller voids of the neutral Ca^II^Cu^II^_6_ porous network. In **Ag**^**+**^**@1** and **Ag**^**0**^**@1**, the thioether chains from methiomox ligand show as detected conformation one of the two crystallographically distinct moieties in a distended conformation towards the pores, and the other one pretty bent with the terminal methyl groups pointing in smaller interstitial voids developing along *a* axis (Fig. 3c,d) and (Figs. S1–S3). Both conformations allow sulfur to target efficiently Ag^+^ ions by S binding sites as confirmed by crystal structure of **Ag**^**+**^**@1** [Ag^+^···S distances of 2.52(2) and 2.54(4) Å]. Nitrate anions, residing within pores, are well stabilized either being coordinated to Ag^+^ metal ions in a chelating motif (Fig. 2c) [Ag^+^···O distances of 2.14(4) and 2.24(9) Å] or by static and non-covalent interactions. Both the Ag^+^ ions residing in the most accessible pores and smaller voids could be reduced to Ag^0^, as confirmed by XPS spectrum of **Ag**^**0**^**@1** (vide infra and Fig. S4). This feature is likely due to visible light-assisted photoreduction process involving Ag^+^ ions.

After the reduction process, the crystal structure of **Ag**^**0**^**@1** unambiguously reveals Ag(0) confined into the pores with metal sites pretty reminiscent of that found in **Ag**^**+**^**@1**. Ag^+^ ions atoms were reduced to Ag_1_^0^ single atoms and Ag_2_ dimers, to give **Ag**^**0**^**@1** (Fig. 3). Ag(0) single atoms are only found in smaller voids (Fig. 3c,d) whereas Ag_2_^0^ dimers are generated in situ from straight Ag^+^ ions reduction and controlled aggregation (being well fixed in pores). Such dimers are retained by sulfur atoms and stabilized through a double binding mode [Ag^0^···S distances of 2.471(9) Å for Ag_1_^0^single atoms and 2.32(3) and 2.732(11) Å for Ag_2_^0^ dimers, featuring an Ag^0^···Ag^0^ separation of 2.59(3) Å] (Fig. 3). The coordinated nitrate anions might play a role in the mechanism of formation of the Ag_2_^0^ dimers in **Ag**^**0**^**@1**, by supporting interactions and by a synergistic effect with the flexible dimethyl thioether chains from the methionine residues, facilitating the approach of silver in bigger hexagonal pores. On the contrary, the reduction process on the silver cations located in the less accessible pores, grew Ag^0^ single atoms still clutched and stabilized by Ag–S interactions.

Apart from the crystal structures, the nature of **Ag**^**+**^**@1** and **Ag**^**0**^**@1** was further established by combining inductively coupled plasm–mass spectrometry (ICP–MS, Table S1, Supporting Information), powder X–ray diffraction (PXRD), Fourier transform infrared spectroscopy (FT-IR) experiments and elemental C, H, S, N and thermo–gravimetric (TGA) analyses, in combination with XPS, which was used to establish the structure and oxidation state of Ag in both **Ag**^**+**^**@1** and **Ag**^**0**^**@1** (see Supporting Information, Figs. S4–S8 and Table S1).

TGA analyses for **Ag**^**+**^**@1** and **Ag**^**0**^**@1** (Fig. S5) allowed to ascertain the water contents for both hybrid MOFs, thus determining their chemical formulas (see experimental section). PXRD patterns of **Ag**^**+**^**@1** and **Ag**^**0**^**@1** (Fig. S6) confirm that bulk (polycrystalline) samples are pure and isostructural to crystals selected for SCXRC in both cases. Moreover they also suggest that open-framework structures remain unaltered after cation insertion (**Ag**^**+**^**@1**) and the reduction process (**Ag**^**0**^**@1**), which is also confirmed by the corresponding FT*-*IR spectra (Fig. S7). In addition, any peaks related to silver metal nanoparticles or oxide crystal structures were not observed in the PXRD pattern of **Ag**^**0**^**@1**, which confirms that only small nanoclusters should be present.

XPS spectra for **Ag**^**+**^**@1** and **Ag**^**0**^**@1** can be observed in Fig. S4. First, XPS spectrum of **Ag**^**+**^**@1** (Fig. S4a) shows the expected two bands at 367.4 and 373.4 eV, which can be unambiguosly attributted to Ag 3d_5/2_ and Ag 3d_3/2_ binding energies, typical of Ag^+^^23,29^. On the other side, XPS spectrum for **Ag**^**0**^**@1** (Fig. S4b) shows the same two bands shifted to 368.2 and 374.2 eV, which suggest that all Ag^+^ cations have been reduced to Ag^0^ atoms. The N_2_ and CO_2_ adsorption isotherms for **Ag**^**+**^**@1** and **Ag**^**0**^**@1**, and also MOF **1** for the sake of comparison, were also studied (Fig. S8). N_2_ adsorption isotherms (Fig. S8a) suggest a certain permanent porosity for **1**, **Ag**^**+**^**@1** and **Ag**^**0**^**@1**, respectively. **Ag**^**+**^**@1** exhibits lower N_2_ adsorption which can be attributed to the presence of Ag^+^ cations and NO_3_^−^ anions occupying the pores. In turn, **Ag**^**0**^**@1** not only exhibits larger N_2_ adsorption than **Ag**^**+**^**@1**, which could be expected as NO_3_^−^ anions are not longer present, but also larger than that of MOF **1**. This behaviour is reproduced in CO_2_ adsorption isotherm (Fig. S8b), which shows a ca. 25% uptake increase for **Ag**^**0**^**@1**. Overall, gas adsorption measurements confirm both that **Ag**^**0**^**@1** possess permanent porosity and also that it is capable to adsorb CO_2_, which are prerequisites for the selected catalytic reaction.

### Catalytic methanation of CO_2_

**Figure 4:**
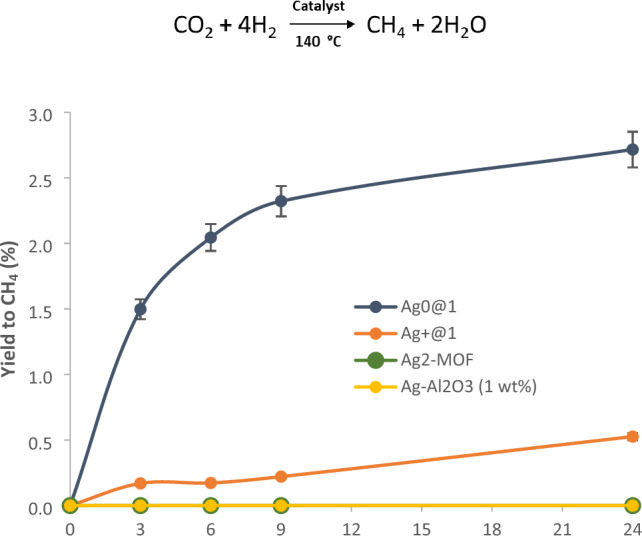
Kinetic results for the methanation of CO_2_ with different solid catalysts. Reaction conditions: 5 bars of the gas mixture N_2_ (internal standard)_,_ CO_2_ and H_2_ (1:1:4), solid catalyst (0.008 mmol of metal, 5 mol% respect to CO_2_), 140 °C. Error bars account for a 5% uncertainty.

The hydrogenation of CO_2_ to methane (Sabatier reaction) has gained attention in the last years as a means to recycle anthropogenic CO_2_ and combat climate change, besides generating renewable methane^26^. Despite the industrial production costs of H_2_ precluded this approach in a first approximation, the raise of a cheap green H_2_ industry could boost this reaction back, with grounded environmental and economic basis^30^. Figure 4 shows the catalytic results for the hydrogenation of CO_2_ with **Ag**^**0**^**@1**. For the sake of comparison, the catalytic behaviour of **Ag**^**+**^**@1**, our previously reported Ag_2_-MOF catalyst^23^, and a house-made sample of Ag–Al_2_O_3_, was also studied. The results show that **Ag**^**0**^**@1** catalyzes the methanation reaction much more efficiently than the other solid catalysts, and with a productivity rate per metal atom comparable to the state-of-the-art Ru–Al_2_O_3_ catalyst under these reaction conditions^27^. Notice here that the reaction temperature (140 °C) was chosen to achieve the maximum reaction rates possible, since Ru–Al_2_O_3_ shows the highest productivity at > 120 °C reaction temperature^27^ and lower temperatures considerable decrease the initial rate of the industrial catalyst^31^. The productivity of the solid catalyst **Ag**^**0**^**@1** was also compared to that of the industrially used Ru on alumina catalyst by calculating the corresponding µmolCO_2_ g^−1^catalyst h^−1^ values (Table S3), under similar reaction conditions^27^. It can be seen that the productivity for **Ag**^**0**^**@1** is not far from the currently used industrial catalyst (68 vs. 93 µmolCO_2_ g^−1^catalyst h^−1^, respectively), which illustrates the remarkable catalytic activity of **Ag**^**0**^**@1**.

Complete hydrogenation of CO_2_ to CH_4_ was found for all catalysts, and neither carbon monoxide, formaldehyde or methanol were not observed in the gas phase, despite conversions are typically < 3%. A kinetic isotopic experiment with D_2_ shows the incorporation of all deuterium atoms in the final methane product (no scrambling with adsorbed water on the solid) and that the breaking of the H–H bond is participating in the limiting step of the hydrogenation reaction, since the kinetic isotopic effect (KIE) = 2.7 (Fig. S9).

$${\text{CO}}_{2} + 4{\text{H}}_{2} \mathop{\longrightarrow}\limits_{{140\;^{ \circ } {\text{C}}}}^{{{\text{Catalyst}}}}{\text{CH}}_{4} + 2{\text{H}}_{2} {\text{O}}$$

**Ag**^**0**^**@1** is easily recovered from the reaction mixture after simple evacuation of the gas reagents, since liquid products are not formed, and can be reused at least three times without severe depletion in the final yield of CH_4_ (Fig. S10). However, an abrupt decrease in catalytic activity occurred after the third use. Characterization of the five-times used solid catalyst by XRD (Fig. S11), XPS (Fig. S12) and high-angle annular dark-field scanning transmission electron microscopy (HAADF–STEM, Fig. S13) confirmed the stability of the Ag_2_ dimer inside the MOF. However, the FT-IR spectrum of this reused catalyst showed a new signal at 1770 cm^−1^, in the typical area for carbon monoxides (CO) species, characteristic of Ag_x_(CO)_x_ species (Fig. S14)^32^. Thus, we can ascribe the poisoning of the catalyst to the formation of CO during reaction, which does not completely evolve to the gas phase but may stay stuck to the Ag^0^ species. The fact that the hydrogenation reaction stops after formation of these Ag_x_(CO)_x_ species, together with the robustness of the **Ag**^**0**^**@1** during reaction, the inactivity of the Ag nanoparticles (Ag–Al_2_O_3_) and the lack of any induction time in the kinetic profile, strongly supports that the Ag species in **Ag**^**0**^**@1** are the truly catalytic active species for the methanation reaction. We placed the recovered catalyst, having the adsorbed CO molecules, under an atmosphere of H_2_, to check if the CO molecules could react, and the analysis of the reaction by GC–MS showed the formation of methane, confirming that the reverse water gas-shift reaction (RWGS) also occurs.

Our previously reported Ag_2_-MOF catalyst^23^, much harder to prepare and without thioether moieties to further stabilize Ag_2_, does not show any catalytic activity for the methanation reaction (see Fig. 4 above), which supports the role of thioether MOF **1** not only for an easy preparation but also for a better catalytic activity and stability of the metal dimer. To check this hypothesis, the analysis of the inactive Ag_2_-MOF material after reaction was accomplished, by PXRD (Fig. S15) and also by HAADF–STEM (Fig. S16). The PXRD analysis (Fig. S15) shows the appearance of peaks at 38° and 44° in the used MOF, which correspond to the (111) and (200) crystallographic planes of Ag NPs. The HAADF–STEM images clearly show the formation of Ag NPs, some of them > 10 nm, confirmed by the corresponding mapping. Notice that Ag NPs are catalytically inactive under our reaction conditions according to the lack of catalytic activity of Ag NPs on alumina (see Fig. 4). These results, together, strongly support the instability of the Ag dimers in the absence of Ag–S interactions within the MOF (compare the new results with the stable **Ag**^**0**^**@1** MOF after reaction, Figs. S11–S14), in other words, the Ag–S bond makes the Ag dimers to be more stable during the methanation reaction.

To further corroborate these conclusions, we performed periodic DFT calculations about the stability of the different Ag species on both **Ag**^**+**^**@1** and **Ag**^**0**^**@1** materials. The results (Figs. S17–S18 and Table S4) show that the isolated Ag_1_ atoms, both in the channels and in the interstitial regions, interact with two S at ⁓ 2.4–2.5 Å, in agreement with the experimental characterization, and that this interaction leaves a net positive charge of < 0.5e on the Ag_1_ atoms. Ag_2_ species in the interstitial region are not stable and break to interact with available S atoms in the surroundings, to have again each Ag atom interacting with two S atoms at ⁓ 2.4–2.5 Å and with a net positive charge of ⁓ 0.5e. In contrast, Ag_2_ dimers are stable in the large channel, with an optimized Ag–Ag distance of ⁓ 2.89 Å and net positive charges of only 0.18 and 0.38e. One of the Ag atoms is interacting with two S atoms at 2.5 and 2.6 Å, and the second Ag atom is only bonded to one S at 2.6 Å, in agreement with the experimental characterization. All these results indicate that the Ag dimers in the channel are energetically stable and that the interstitial Ag atoms prefer to be in single cationic form. The new computational results in combination with the experimental results confirm that the Ag cations located in the less accessible pores (i.e. interstitial) are not catalytically active, since these interstitial Ag atoms are similarly present and stable in both the active **Ag@1** and inactive Ag_2_-MOF, but only the former catalyzes the methanation reaction. Thus, by substration, the interstitial Ag atoms should not be active for the reaction. Overall, these results reflect the high sensitiveness of the catalysed reaction to the metal atomicity, in this case to just a metal dimer, in line with classical postulates for this type of metal catalysis^33,34^.

## Conclusions

Ag_2_(0) dimers are prepared and stabilized in methioMOF **1**, and act as a solid catalyst for the methanation reaction of CO_2_. Selectivity towards CH_4_ is complete under mild reaction conditions, and the new solid catalyst can be easily recovered and reused three times without severe catalytic depletion. These results open the way to employ these ultrasmall Ag clusters as catalysts in hydrogenation reactions. The findings here shown constitute a new example of how MOFs can host metal species otherwise unstable and difficult to synthesize, to be efficiently used in catalysis.

## Supporting Information (SI) available

Experimental and characterization details, additional Tables S1–S4 and Figures S1–S18. Compounds **Ag**^**+**^**@1** and **Ag**^**0**^**@1 **have been assigned in the Cambridge Structural Database the deposition numbers CCDC 2237789 and 2237790, respectively.

## Supplementary Information

Supplementary Information.

## Data Availability

The datasets generated during and/or analysed during the current study are available from the corresponding author on reasonable request.
