# Supplementary material for: Ag2(0) dimers within a thioether-functionalized MOF catalyze the CO2 to CH4 hydrogenation reaction
Source: Sci Rep. 2023 Jun 27;13:10376. doi: 10.1038/s41598-023-37600-4 (PMC10300047; doi:10.1038/s41598-023-37600-4)
Supplement: Supplementary file 1 — Supplementary Information. [file 41598_2023_37600_MOESM1_ESM.pdf]

Supporting Information

$Ag_2(0)$ dimers within a thioether−functionalized MOF catalyze the CO_2 to CH_4 hydrogenation reaction

Yongkun Zheng,[a] Nuria Martín,[b] Mercedes Boronat,[a] Jesús Ferrando−Soria,[b] Marta Mon,[a]* Donatella Armentano,[c]* Emilio Pardo[b]* and Antonio Leyva–Pérez[a]*

[a] *Instituto de Tecnología Química (UPV–CSIC), Universidad Politècnica de València–Consejo Superior de Investigaciones Científicas, Avda. de los Naranjos s/n, 46022 Valencia, Spain;* [b] *Departamento de Química Inorgánica, Instituto de Ciencia Molecular (ICMol), Catedrático José Beltrán Martínez, 2, Universidad de Valencia, 46980 Paterna, Valencia, Spain;* [c] *Dipartimento di Chimica e Tecnologie Chimiche (CTC), Università della Calabria, Rende 87036, Cosenza, Italy.*

Corresponding authors´ e−mails: marmoco@itq.upv.es; donatella.armentano@unical.it; emilio.pardo@uv.es; anleyva@itq.upv.es.

<u>**Table of contents**</u>

Experimental Section p. S2−S6

Supporting Tables (Table S1−S4) p. S7−S8

Supporting Figures (Figures S1 – S18) p. S9−S26

Supporting References p. S27

Experimental Section

Materials. All chemicals were of reagent grade quality. They were purchased from commercial sources and used as received. {$Ca^{II}Cu^{II}_6$[(*S,S*)−methox]$_3$(OH)$_2$(H$_2$O)} · 16H$_2$O (**1**) was prepared as previously reported [S1].

($Ag^{I}NO_3$)$_3$@{$Ca^{II}Cu^{II}_6$[(*S,S*)–methox]$_3$(OH)$_2$(H$_2$O)} · 13H$_2$O (Ag$^+$@1): Well–formed hexagonal green prisms of **2**, which were suitable for X–ray diffraction, were obtained by soaking crystals of MOF **1** (*ca.* 25 mg, 0.015 mmol) in a H_2O/CH_3OH (1:1) solution of $AgNO_3$ (0.015 mmol) for 1 h in the darkness. The process was repeated ten times to ensure the maximum loading of $AgNO_3$. Crystals were washed with a H_2O/CH_3OH (1:1) solution several times, isolated by filtration on paper, air–dried and stored in the darkness. Anal.: calcd for $C_{36}Cu_6CaH_{78}Ag_3S_6N_9O_{43}$ (2262.4): C, 19.11; H, 3.47; S, 8.50; N, 5.57%. Found: C, 18.99; H, 3.41; S, 8.51; N, 5.62%. IR (KBr): ν = 1604 cm^{-1} (C=O).

Moreover, a gram−scale procedure was also carried out by using the same synthetic procedure but using a higher amount of a polycrystalline sample of MOF **1** (2 g, 1.1 mmol), which were suspended a H_2O/CH_3OH (1:1) solution of $AgNO_3$ (1.1 mmol) for 1 hour under a mild stirring in the darkness. The process was repeated 10 times. Finally, the product was collected by filtration, washed with a H_2O/CH_3OH (1:1) solution, air–dried and stored in the darkness. Anal.: calcd for $C_{36}Cu_6CaH_{78}Ag_3S_6N_9O_{43}$ (2262.4): C, 19.11; H, 3.47; S, 8.50; N, 5.57%. Found: C, 19.11; H, 3.37; S, 8.59; N, 5.45%. IR (KBr): ν = 1607 cm^{-1} (C=O).

(Ag^0_2)(Ag^0)@{$Ca^{II}Cu^{II}_6$[(*S,S*)−methox]$_3$(OH)$_2$(H$_2$O)} · 14H$_2$O (Ag^0@1): The same procedure was applied, with the same successful results to both, crystals (*ca.* 25 mg) and a powder polycrystalline sample of **Ag$^+$@1** (*ca.* 2 g). They were suspended in H_2O/CH_3CH_2OH (1:6) solutions to which $NaBH_4$, divided in 15 fractions (0.4 mmol of $NaBH_4$ per mmol of MOF each), were added progressively in the space of 72 hours. Each fraction was allowed to react for 1.5 hour. After this period, samples were gently washed with a H_2O/CH_3OH solution and filtered on paper. Anal.: calcd for $C_{36}Cu_6CaH_{80}Ag_3S_6N_6O_{39}$ (2094.4): C, 20.64; H, 3.85; S, 9.19; N, 4.01%. Found: C, 20.61; H, 3.76; S, 9.21; N, 3.99%. IR (KBr): ν = 1601 cm^{-1} (C=O).

Physical Techniques. Elemental (C, H, N) and ICP−MS analyses were performed at the Microanalytical Service of the Universitat de València. FT–IR spectra were recorded on a JASCO FT/IR−4700 spectrophotometer. The thermogravimetric analysis was performed on crystalline samples under a dry N_2 atmosphere with a Mettler Toledo TGA/STDA 851e thermobalance operating at a heating rate of 10 ºC min^{-1}. GC analyses were performed after extracting samples of the reaction with a Hamilton SampleLock gas syringe. Reaction products were analyzed with an Agilent micro−GC (Molsieve 5A column with Ar as carrier gas) by comparison with isolated commercial products.

Gas adsorption measurements. The N_2 adsorption–desorption isotherms at 77 K were carried out on crystalline samples of **Ag$^+$@1** and **Ag^0@1** with a Belsorp Mini X instrument. Samples were evacuated at 70 ºC during 24 h under 10^{-6} Torr prior to their analysis.

X–ray Powder Diffraction Measurements. Polycrystalline samples of **Ag$^+$@1** and **Ag^0@1,** and also after catalysis for **Ag^0@1**, were introduced into 0.5 mm borosilicate capillaries prior to being mounted and aligned on an Empyrean PANalytical powder diffractometer, using Cu Kα radiation (λ = 1.54056 Å). For each sample, five repeated measurements were collected at room temperature (2θ = 2–60°) and merged in a single diffractogram.

X–ray photoelectron spectroscopy (XPS) measurements. Samples of **Ag$^+$@1** and **Ag^0@1** were prepared by sticking, without sieving, the samples onto a molybdenum plate with scotch tape film, followed by air drying. Measurements were performed on a K−Alpha™ X−ray Photoelectron Spectrometer (XPS) System using a monochromatic Al K(alpha) source (1486.6 eV). As an internal reference for the peak positions in the XPS spectra, the C1s peak has been set at 284.8 eV.

Microscopy measurements. Scanning Electron Microscopy (SEM) elemental analysis was carried out for **Ag$^+$@1** and **Ag^0@1**, using a HITACHI S−4800 electron microscope coupled with an Energy Dispersive X–ray (EDX) detector. Data was analyzed with QUANTAX 400. The images of the **Ag^0@1** before and after methanation reaction were obtained on a Jeol JEM−F2100 microscope operated at 200 kV in dark field scanning transmission electron microscopy (DF−STEM mode).

X–ray crystallographic data collection and structure refinement. Crystals of **Ag^+@1** and **Ag_2@1**with 0.14 x 0.12 x 0.12 mm and 0.08 x 0.08 x 0.06 as dimensions were selected and mounted on a MITIGEN holder in Paratone oil and very quickly placed on a nitrogen or liquid helium stream cooled at 90 or 45 K for **Ag^+@1** and **Ag_2@1**, respectively to avoid the possible degradation upon dehydration. Diffraction data for **Ag^+@1** were collected on a Bruker−Nonius X8APEXII CCD area detector diffractometer, using graphite−monochromated Mo−Kα radiation (λ = 0.71073 Å) whereas for **Ag_2@1**, were collected using synchrotron radiation at CRISTAL beamline of the SOLEIL (λ = 0.67165 Å). The data were processed through SAINT[S2] and CrysAlisPro [S3], reduction and SADABS[S4] multi–scan absorption software. The structures were solved with the SHELXS structure solution program, using the Patterson method. The model was refined with version 2018/3 of SHELXL against F^2 on all data by full–matrix least squares [S5,S6].

As reported in the main text, the robustness of the 3D network, allowed the resolution of the crystal structure of both **Ag^+@1** and **Ag_2@1**, adsorbates, being their crystals suitable for X–ray diffraction, even over one– and two–step process, after a crystal–to–crystal transformation. For these reasons it is reasonable to observe a diffraction pattern sometimes affected by expected internal imperfections of the crystals [likely at the origin of some Alert level A for **Ag^+@1** and **Ag_2@1**in checkcifs related to U(eq) value of some atoms] and thus a quite expected difficulty to perform a perfect correction of anisotropy, mainly affected by highly flexible thioether chains as terminal moiety (vide infra).

In both samples, all non–hydrogen atoms of the MOF network, except some dynamically disordered fragments of the ethylenethiomethyl chains of the methox ligand, NO_3^- anions in **Ag^+@1**, and the thermally disordered Ag^+ and Ag^0 atoms, were refined anisotropically. The use of some C−C and C−S bond lengths restrains as well as Ag−$O_{NITRATE}$ (**Ag^+@1**) and Ag−S (**Ag_2@1**), during the refinements, has been reasonable imposed and related to extraordinary flexibility of ethylenethiomethyl chains of the methox ligand that are dynamic components of the frameworks (see Figures S3). It is likely depending on the large pore's size of the frameworks (FLAT, DFIX, DANG, SIMU, DELU). Disordered sites for atoms C5S, C6S, S2 in refinement of **Ag_2@1**, belonging to the thioether chains, after a free all variables refinement, have been modelled with fixed parameters.

The occupancy factors of Ag ions/atoms have been defined in agreement with SEM and ICP–MS results [0.3333 for Ag1 and 0.6666 for Ag2 in **Ag^+@1** and 0.3333 for Ag_1, Ag_2 and Ag_3 in **Ag_2@1**, in agreement with stoichiometry after the reduction process.

The solvent molecules were highly disordered but, even if not all the ones detected by TGA analysis, have been modelled. In the refinement of **Ag_2@1** also confined NO_3^- anions have been found from the ΔF map, and refined with restrains of N−O bond lengths and O−N−O angles, the quite large channels featured by this series of MOFs likely account for the detected thermal disorder.

A summary of the crystallographic data and structure refinement for the two compounds is given in Table S2. The comments for the alerts A and B are reported in the CIFs using the validation response form (vrf). CCDC reference numbers are CCDC 2237789 and 2237790 for **Ag^+@1** and **Ag_2@1**, respectively.

The final geometrical calculations on free voids and the graphical manipulations were carried out with PLATON[S7] implemented in WinGX [S8], and CRYSTAL MAKER[S7] programs, respectively.

Synthesis of Ag on alumina. Commercial aluminum oxide (1 g) was impregnated with a solution of silver nitrate in water (15.7 mg in 1.9 ml), and the mixture was dried in an oven at 100 °C overnight to obtain Ag–Al_2O_3 (1 wt%).

Typical procedure for the catalytic methanation reaction. The reactions were performed in a 7 mL glass vial equipped with a valve and a manometer. The solid catalyst (0.008 mmol of metal in each case, 5 mol% respect to CO_2) was added, and the glass vial was closed and purged for 3 times with a gas mixture of N_2 (internal standard), CO_2 and H_2 (1:1:4), for three times. Then, the gas mixture was added through the valve, and pressurized to 5 bars. Reactions were set at 140 ºC for 24 h. Samples were extracted using a Hamilton SampleLock gas syringe and reaction products analyzed by micro−GC.

Reuse of the Ag@1 catalyst. The general reaction procedure above was followed. After 24 h reaction time, the gas reagents were evacuated, the glass vial was purged with the gas mixture N_2, CO_2 and H_2 (1:1:4) for three times, and the reaction was carried out again under the same reaction conditions.

Isotopic experiment. The reactions were performed in a 7 mL glass vial equipped with a valve and a manometer. Solid catalyst (0.0048 mmol of silver) was added, and the glass vial was closed and purged three times with a gas mixture of N_2 (internal standard), CO_2 and H_2 or D_2 (1:1:4). Then, the gas mixture was added through the valve, pressurizing to 3.5 bars. Reactions were set at 140 ºC for 5 h. Samples were extracted using a Hamilton SampleLock gas syringe and reaction products analyzed by micro−GC.

Computational details. Periodic density functional theory (DFT) calculations were performed with the Vienna Ab-initio Simulation Package (VASP) code [S9], using the Perdew-Burke-Ernzerhof (PBE) exchange-correlation functional [S10]. The valence density was expanded in a plane wave basis set with a kinetic energy cutoff of 600 eV, and the effect of the core electrons in the valence density was taken into account by means of the projected augmented wave (PAW) formalism [S11]. Integration in the reciprocal space was carried out at the Γ k-point of the Brillouin zone. During geometry optimizations, the positions of all atoms in the system were allowed to relax without restrictions. Atomic charges were estimated using the theory of atoms in molecules (AIM) of Bader [S12]. The MOF was described by means of a hexagonal unit cell with parameters a = b =18.057, c = 12.800, containing 2 Ca, 12 C, 12 S, 12 N, 60 C, 42 O and 80 H atoms. One Ag atom (Ag_1) and one Ag dimer (Ag_2) were placed in two different positions, in the channel and in the interstitial region, and the geometry of the resulting system was optimized without restrictions.

Supporting Tables

Table S1. Selected data from the ICP–MS[a] and SEM/EDX[b] analyses.

		**Ag^+@1**		
**Metal**	*% mass*[a]	*Metal stoichiometry*[a]	*% mass*[b]	*Metal stoichiometry*[b]
**Cu**	16.85	6.00	16.89	6.00
**Ca**	1.78	1.01	1.67	0.94
**Ag**	14.28	2.99	14.39	3.02
		**Ag^0@1**		
**Metal**	*% mass*[a]	*Metal stoichiometry*[a]	*% mass*[b]	*Metal stoichiometry*[b]
**Cu**	17.69	6.00	17.59	6.00
**Ca**	1.84	0.99	1.67	0.90
**Ag**	15.03	2.00	14.55	1.94

[a] Solid samples were digested with 0.5 mL of HNO_3 69% at 60°C for 4 hours followed by the addition of 0.5 mL of HCl 37% and digestion 80°C for 1 hour. The metal stoichiometry was calculated in both cases with respect to Cu values.

Table S2. Summary of Crystallographic Data for **Ag^+@1** and **Ag_2@1**.

Compound	**Ag^+@1**	**Ag_2@1**
Formula	$C_{36}Cu_6CaH_{78}Ag_3S_6N_9O_{43}$	$C_{36}Cu_6CaH_{80}Ag_3S_6N_6O_{39}$
M (g mol^{-1})	2262.36	2158.35
λ (Å)	0.71073	0.67165
Crystal system	Hexagonal	Hexagonal
Space group	$P6_3$	$P6_3$
a (Å)	17.3731(13)	17.1791(6)
c (Å)	13.7165(10)	13.6951(5)
V (Å^3)	3585.3(6)	3500.2(3)
Z	2	2
ρ_{calc} (g cm^{-3})	2.096	2.048
μ (mm^{-1})	2.894	2.954
T (K)	90	45
θ range for data collection (°)	1.353 to 24.994	2.645 to 24.487
Completeness to θ = 25.0	100%	98%
Measured reflections	31299	10659
Unique reflections (Rint)	4217(0.1051)	3852(0.0736)
Observed reflections [$I > 2\sigma(I)$]	2773	2817
Goof	1.064	1.150
R^a [$I > 2\sigma(I)$] (all data)	0.1125 (0.1693)	0.0984 (0.1289)
wR^b [$I > 2\sigma(I)$] (all data)	0.2999 (0.3577)	0.2784 (0.3020)

^{a} $R = \sum(|F_o| - |F_c|)/\sum|F_o|$. ^{b} $wR = [\sum w(|F_o| - |F_c|)^2/\sum w|F_o|^2]^{1/2}$.

Table S3. Productivity (μmolCO_2·g^{-1}catalyst·h^{-1}) for different methanation catalysts in the literature (see ref. [27] in the text) under the optimized methanation reaction conditions employed in this work.

**Entry**	**Catalyst**	**Productivity (μmolCO_2·g^{-1}catalyst·h^{-1})**
1	Ru on alumina	93
2	Pt on alumina	7
3	**Ag^+@1**	68
4	**Ag^0@1**	8

Table S4. DFT results for **Ag^+@1** and **Ag_2@1** (see also Figures S17-S18).

	**Ag^+@1**		**Ag_2@1**			
	channel	interstitial	channel		interstitial	
qAg	0.491	0.467	0.182	0.381	0.491	0.508
rAg-Ag			2.895		8.403	
rAg-S	2.435	2.413	2.490	2.578	2.381	2.399
	2.506	2.431	2.604		2.509	2.427
rAg-O	2.313	2.649	2.506		2.352	2.556

Supporting Figures

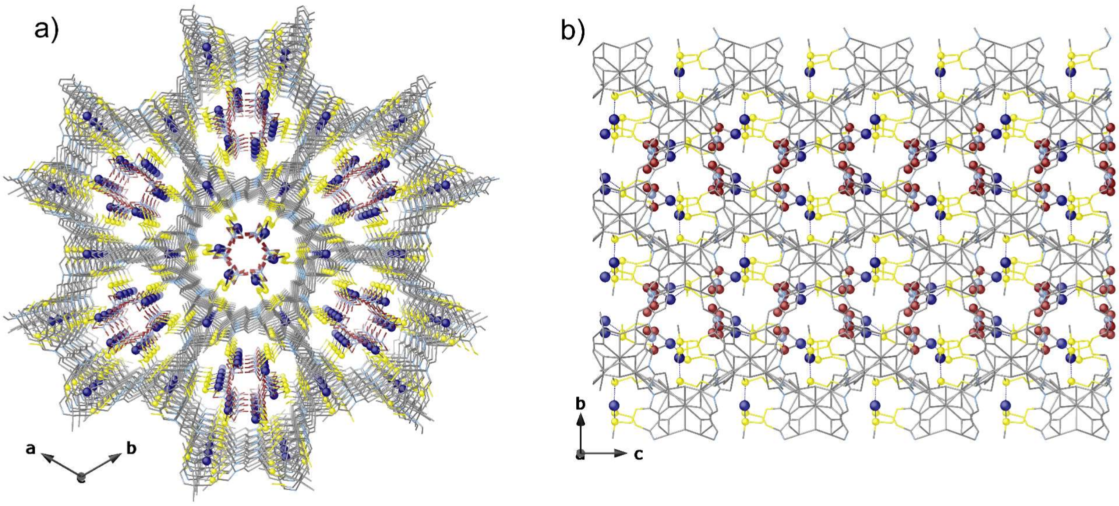

Figure S1. Perspective view of **Ag^{+}@1** crystal structure along *c* (a) and *a* axis (b). Yellow and blue spheres represent S and Ag atoms whereas all the porous network is depicted as gray sticks. Sky blue and red spheres represent N and O atoms of nitrate anions. Blues dashed lines represent the Ag···S interactions.

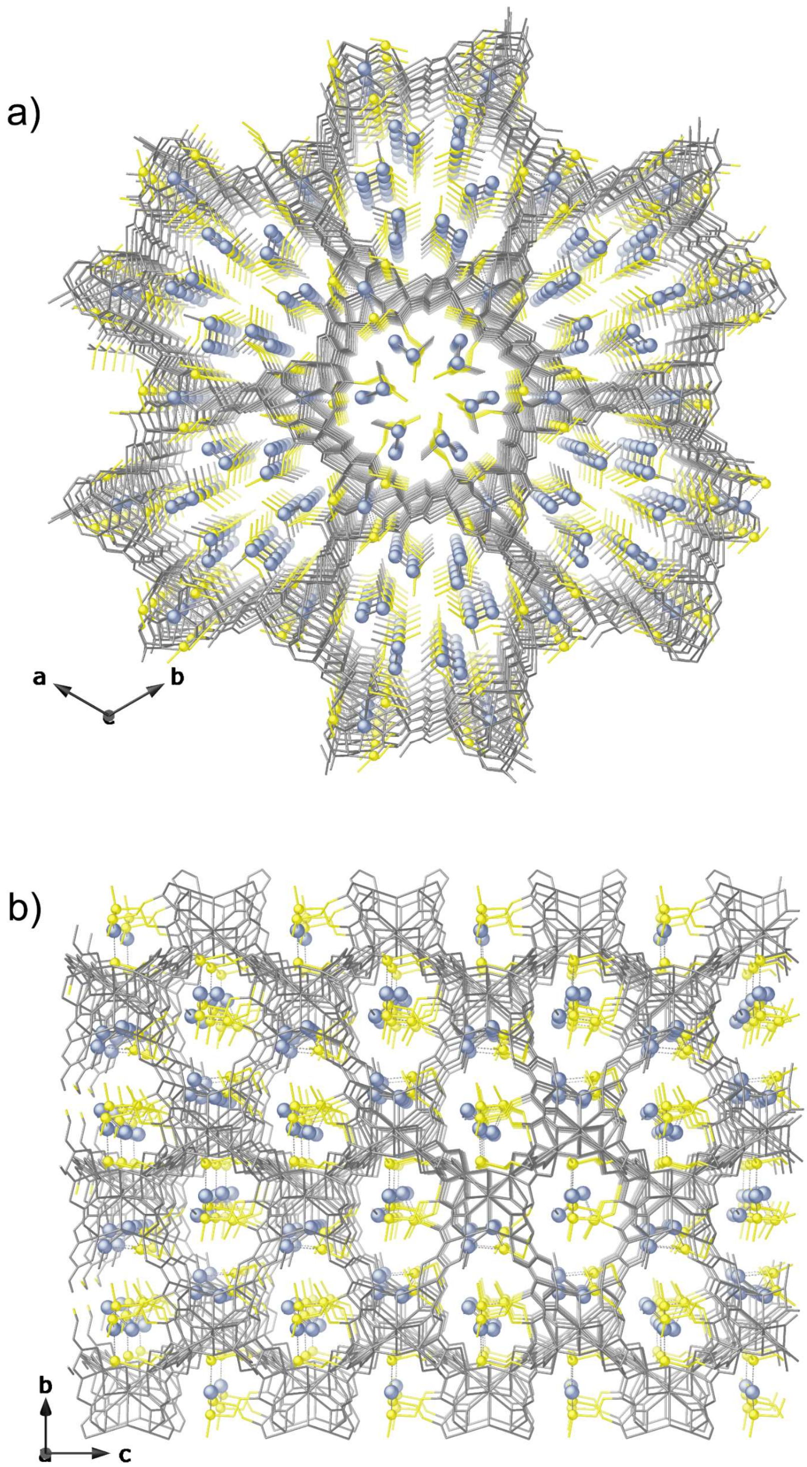

Figure S2. Perspective view of **Ag⁺@1** crystal structure along *c* (a) and *a* axis (b). Yellow and light blue spheres represent S and Ag0 atoms, respecively whereas all the porous network is depicted as gray sticks except for methionine arms represented as yellow sticks. Light blue dashed lines represent the Ag···S interactions.

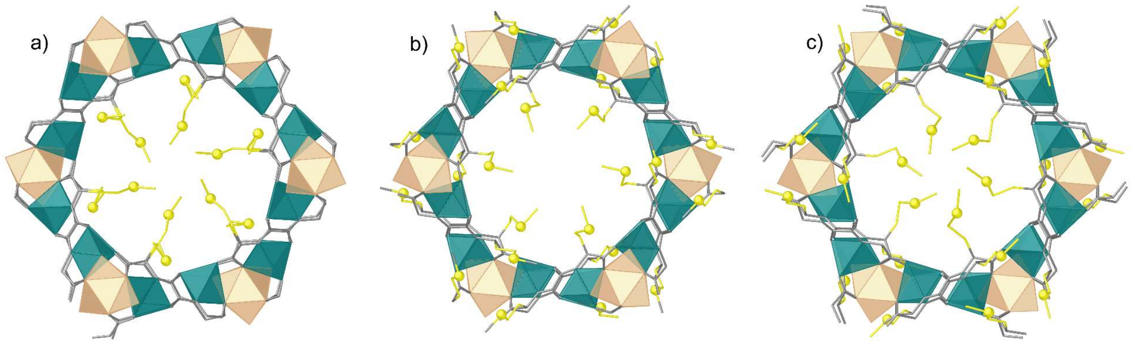

Figure S3. Comparison of the different conformations of methionine moieties in pore's for crystal structures **1** (a), **Ag^{+}@1** (b) and **Ag_2@1** (c) showing the high flexibility of the systems, confined in channels. [The silver ions/atoms for **Ag^{+}@1** (b) and **Ag_2@1** together with NO_3^- anions and solvent water molecules have been omitted for clarity]. The confinement of one of the two crystallographically distinct arms in smaller voids developing along *a* axis is evident only in **Ag^{+}@1** and **Ag_2@1**. It is likely due to the presence of most hindering NO_3^- and Ag^+ ions, or Ag^0 and Ag^0_2 species, respectively. Color scheme: Sulfur, yellow spheres; calcium, pastel orange polyhedra; copper, dark cyan polyhedral; atoms from the ligand, gray sticks.

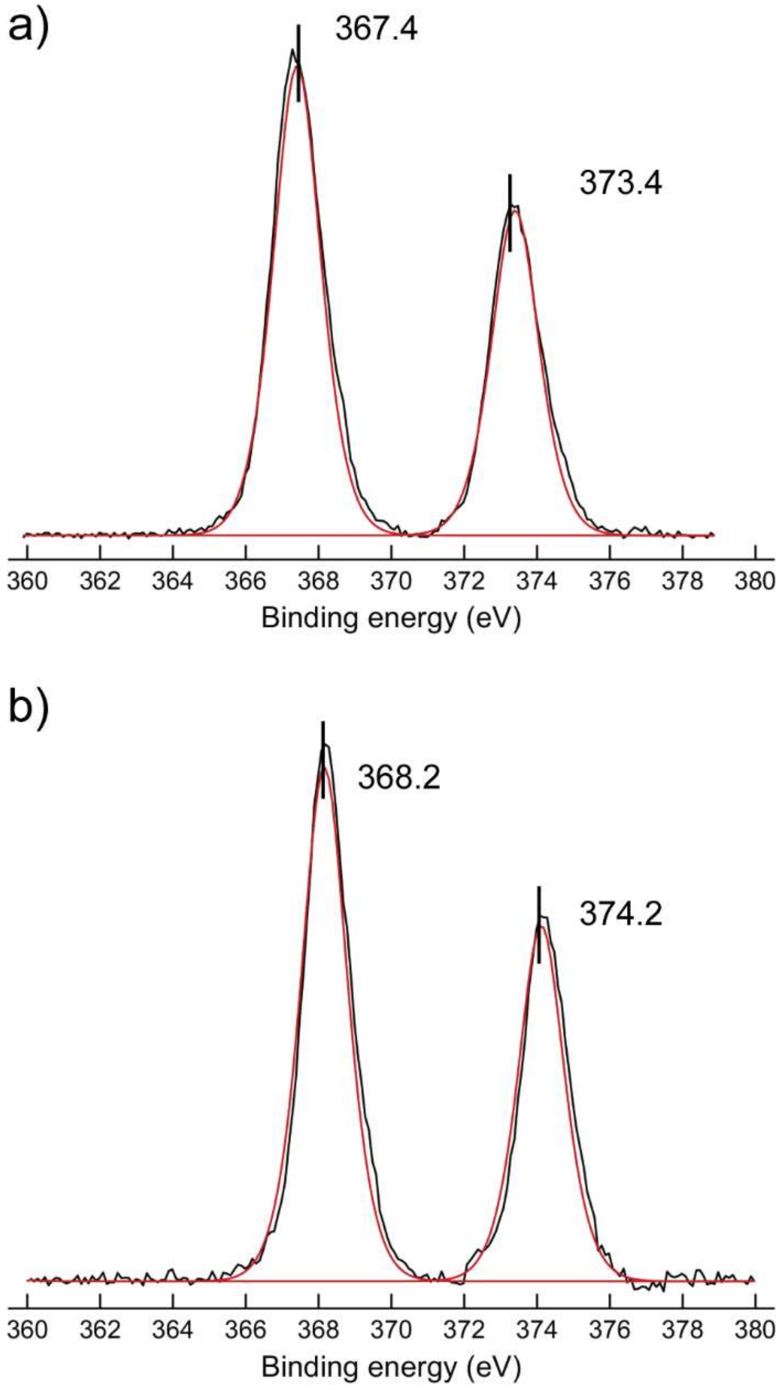

Figure S4. X–ray photoelectron spectroscopy (XPS) of **Ag$^+$@1** (a) and **Ag^0@1** (b).

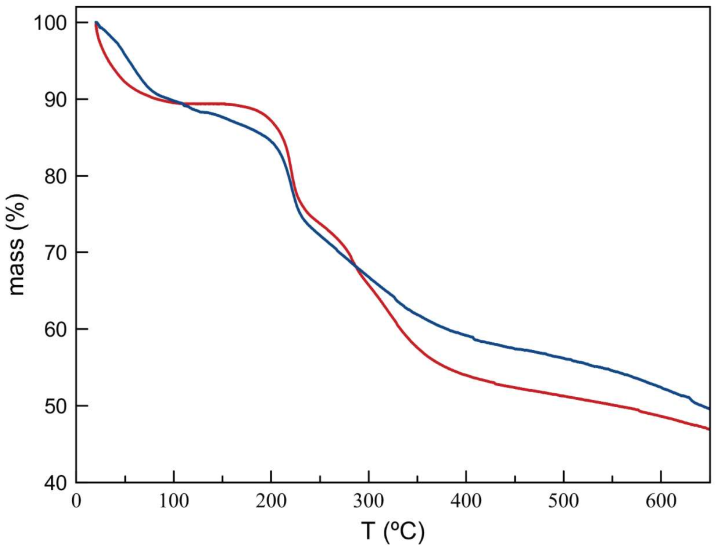

Figure S5. Thermo–Gravimetric Analyses (TGA) of **Ag^{+}@1** (blue) and **Ag^{0}@1** (red) under dry N_2 atmosphere.

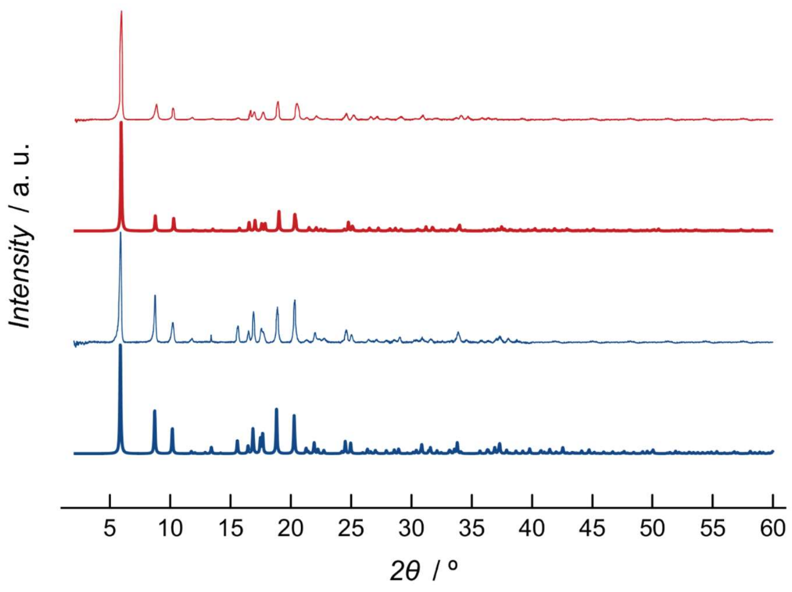

Figure S6. Calculated (bold lines) and experimental (solid lines) PXRD pattern profiles of **Ag^{+}@1** (blue) and **Ag^{0}@1** (red) in the 2θ range 2.0–60.0°.

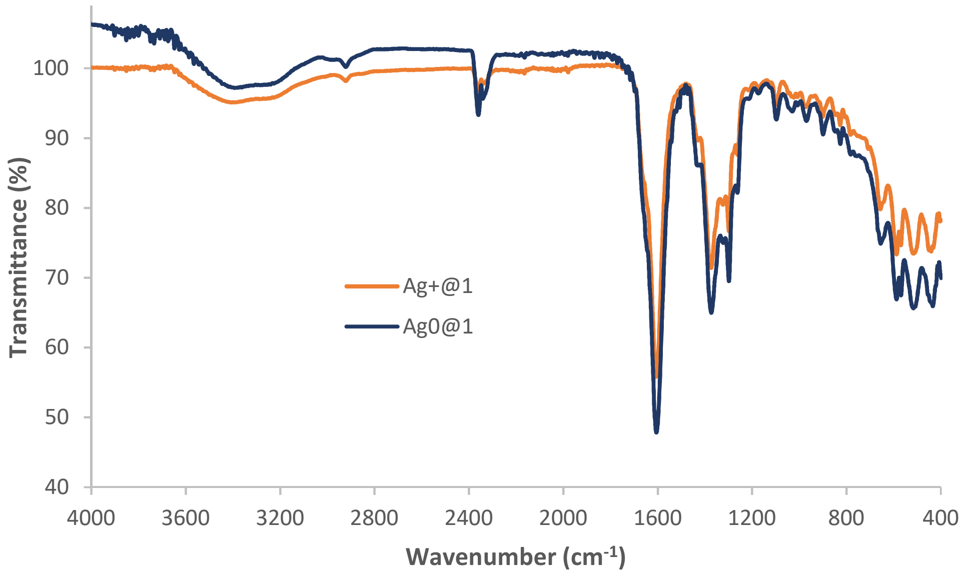

Figure S7. FT−IR spectra of **Ag^{+}@1** (orange) and **Ag^{0}@1** (blue).

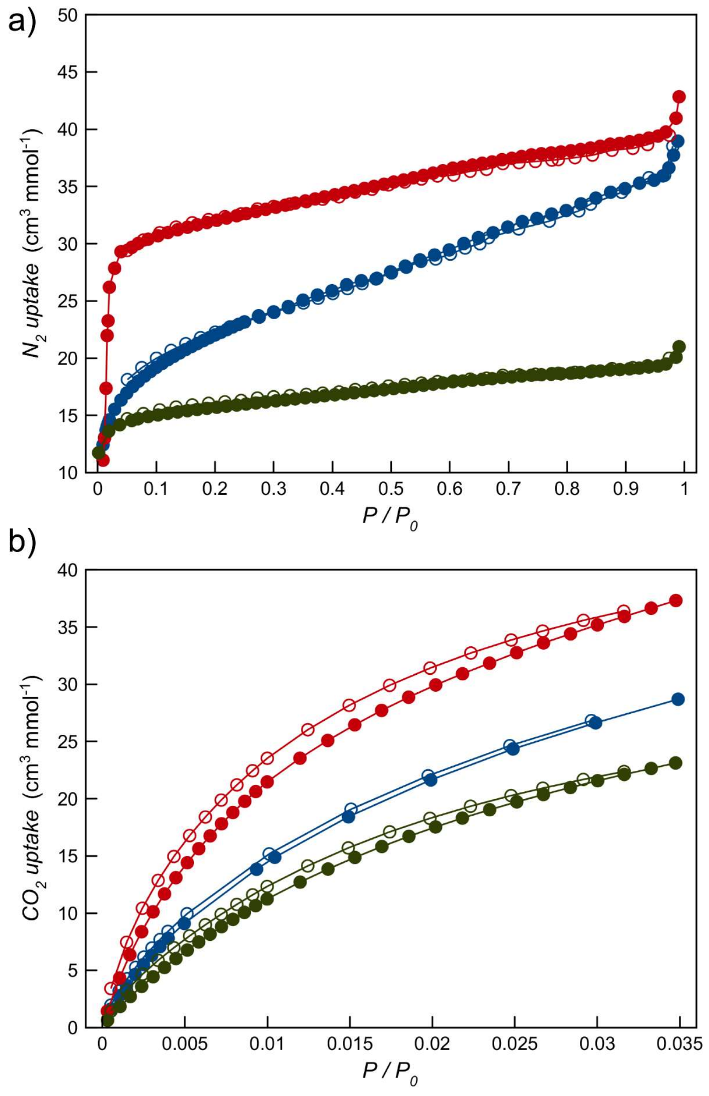

Figure S8. (a) N_2 (77 K) and (b) CO_2 (273 K) adsorption isotherms for the activated compounds **1** (blue), **Ag⁺@1** (green) and **Ag⁰@1** (red). Filled and empty symbols indicate the adsorption and desorption isotherms, respectively. The samples were activated at 70 °C under reduced pressure for 24 h prior to carry out the sorption measurements.

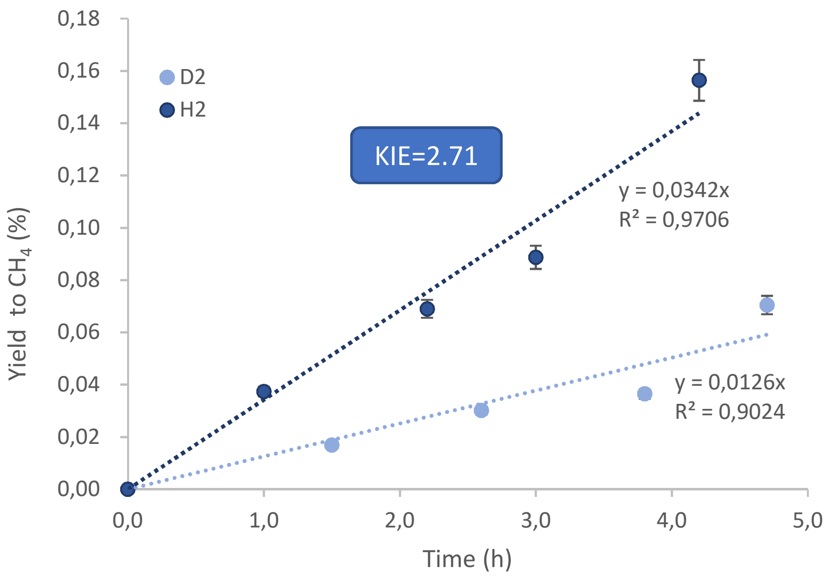

Figure S9. Results for methanation reaction of CO_2 with H_2 or D_2 using **Ag^0@1** as a catalyst. Reaction conditions: 3.5 bars of the gas mixture N_2 (internal standard), CO_2 and H_2 (1:1:4) or N_2, CO_2 and D_2 (1:1:4), **Ag^0@1** (0.0048 mmol of silver), 140 ºC. Error bars account for a 5% uncertainty.

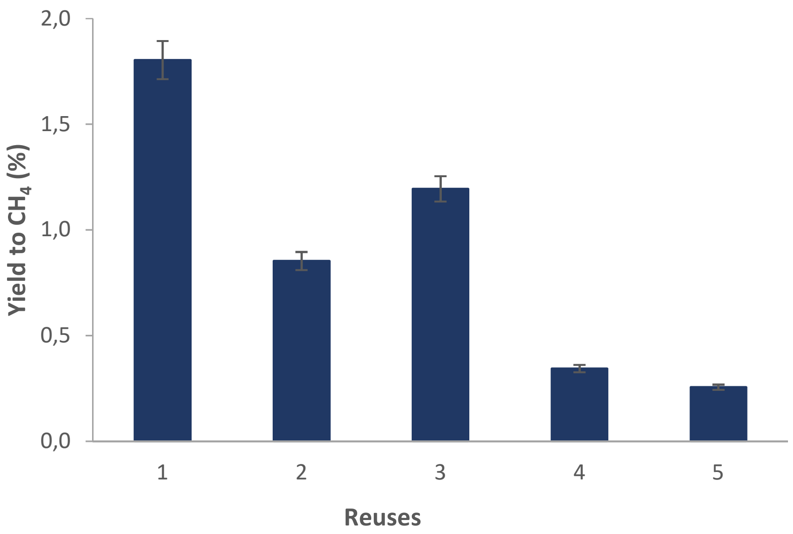

Figure S10. Catalytic reuses of **Ag^0@1** as a catalyst for the methanation reaction of CO_2. Reaction conditions: 5 bars of the gas mixture N_2 (internal standard), CO_2 and H_2 (1:1:4), **Ag^0@1** (0.008 mmol of silver, 5 mol% respect to CO_2), 140 ºC. Error bars account for a 5% uncertainty.

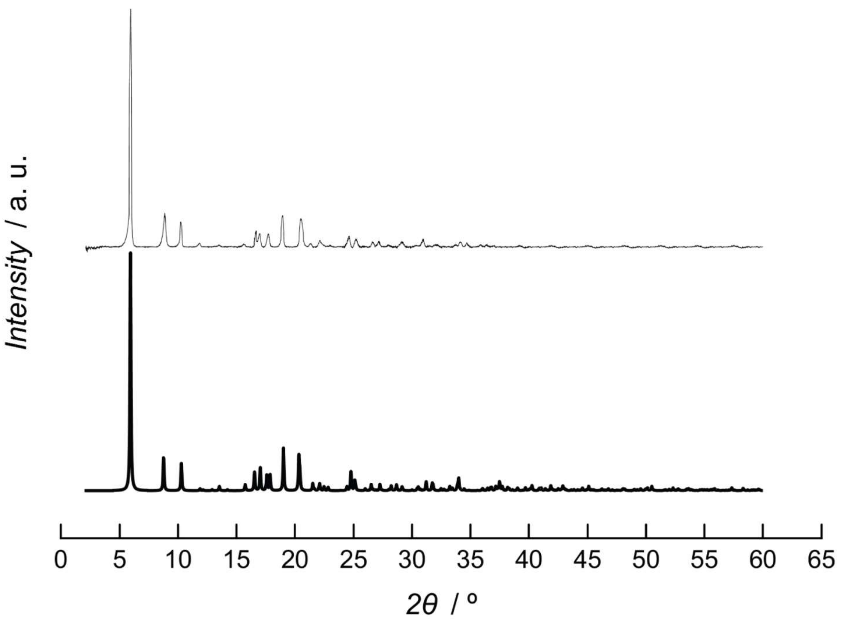

Figure S11. Calculated (bold line) and experimental (solid line) PXRD pattern profiles of **Ag^0@1** in the 2θ range 2.0–60.0°, after catalysis.

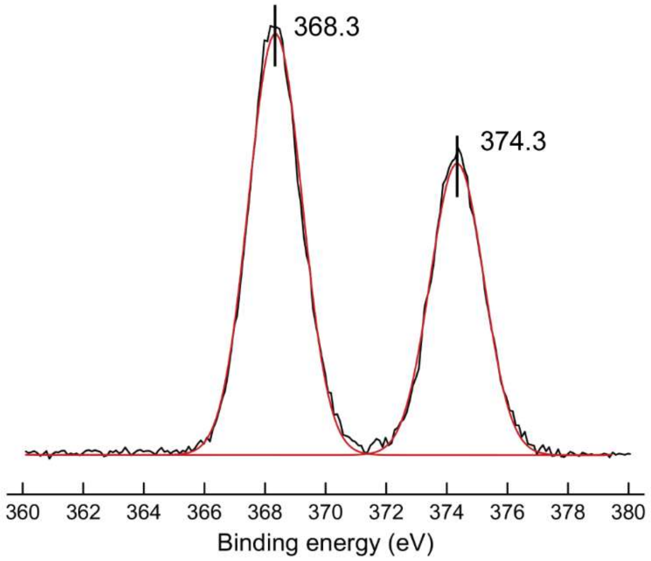

Figure S12. X–ray photoelectron spectroscopy (XPS) of **Ag^0@1** after catalysis.

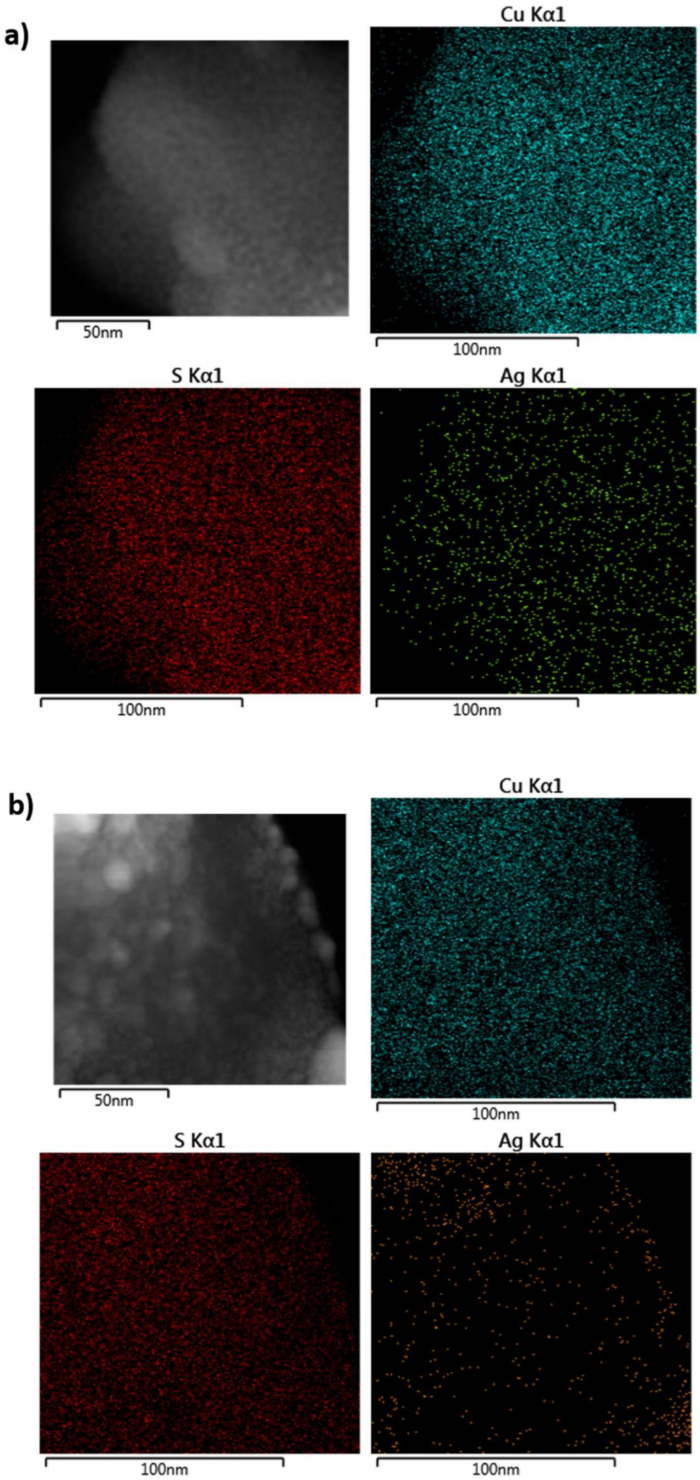

Figure S13. High−angle annular dark−field scanning transmission electron microscopy (HAADF−STEM) images and particle mapping analysis of the **Ag^0@1** catalyst (a) before and (b) after methanation reaction.

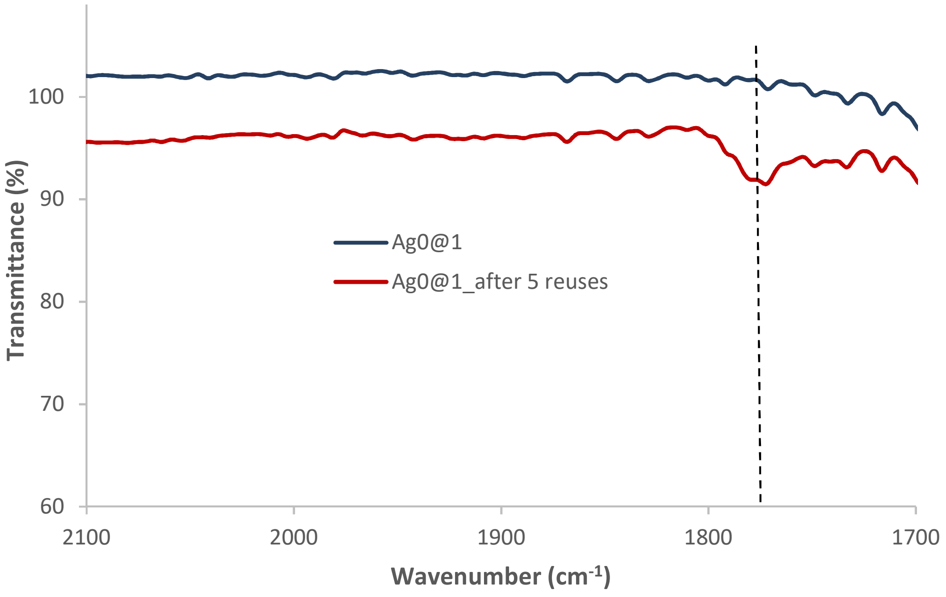

Figure S14. FT−IR spectra in the 2100−1700 cm^{-1} region of **Ag^0@1** before the methanation reaction (blue) and after 5 uses (red). The band associated to $Ag_x(CO)_x$ species has been pointed out.

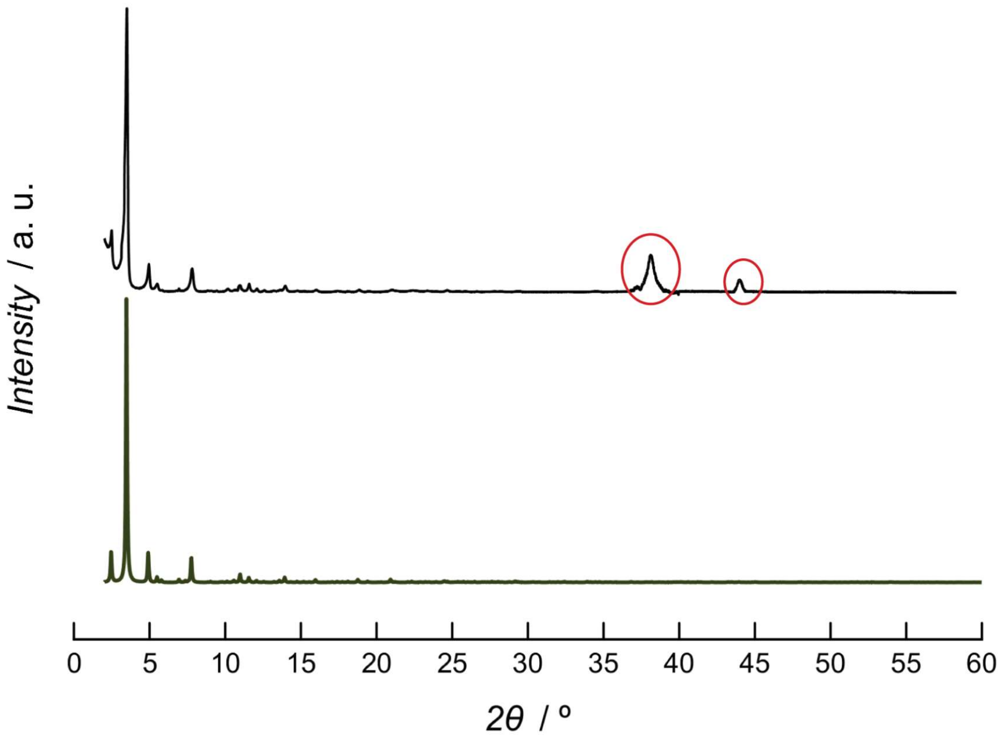

Figure S15. Experimental PXRD pattern profile of the inactive Ag_2−MOF before (bottom) and after the methanation reaction (top), where the presence of peaks corresponding to the (111) and (200) crystallographic planes of Ag nanoparticles is clearly seen (red circles).

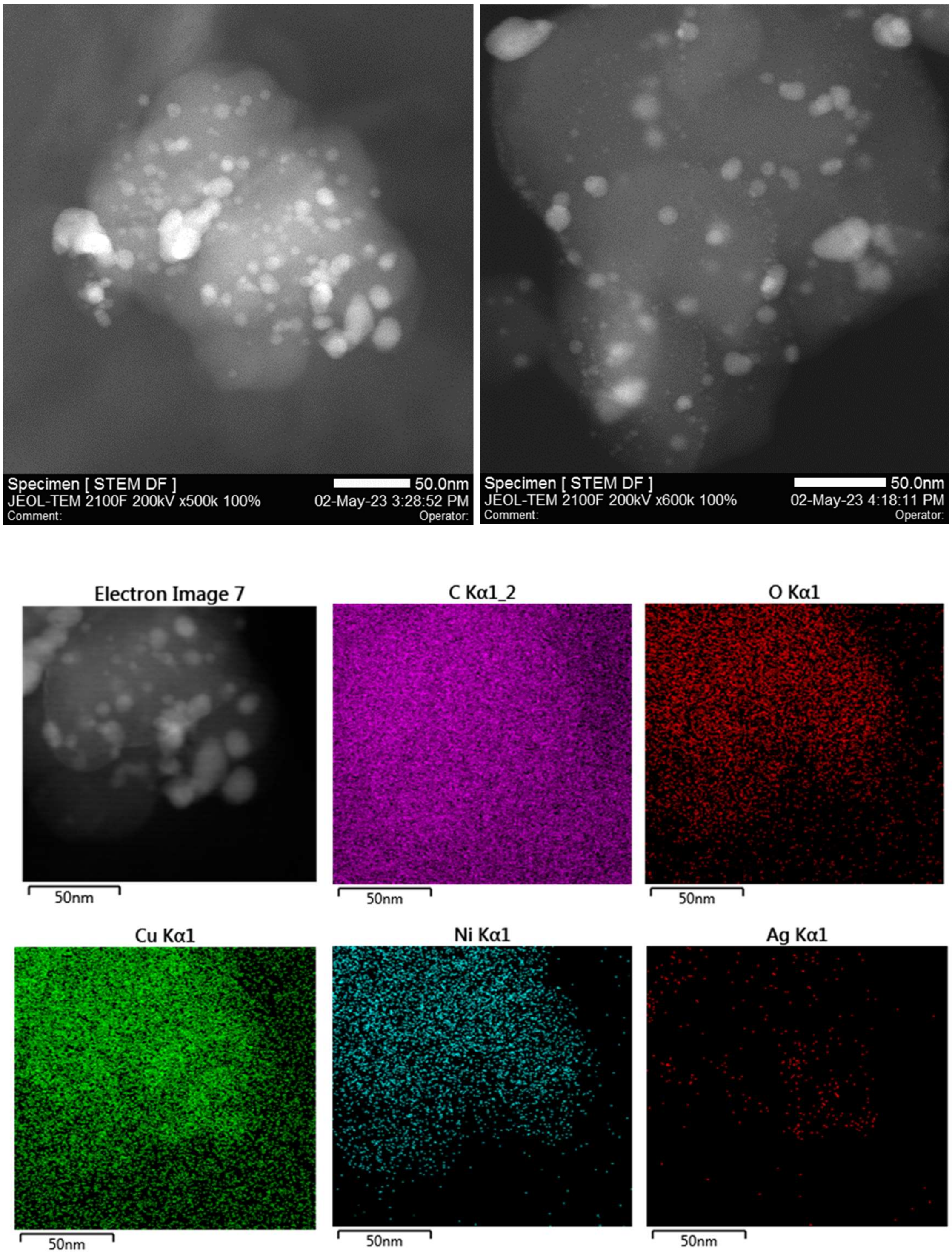

Figure S16. High−angle annular dark−field scanning transmission electron microscopy (HAADF−STEM) images (top) and particle mapping analysis (bottom) of the inactive Ag_2−MOF after the methanation reaction.

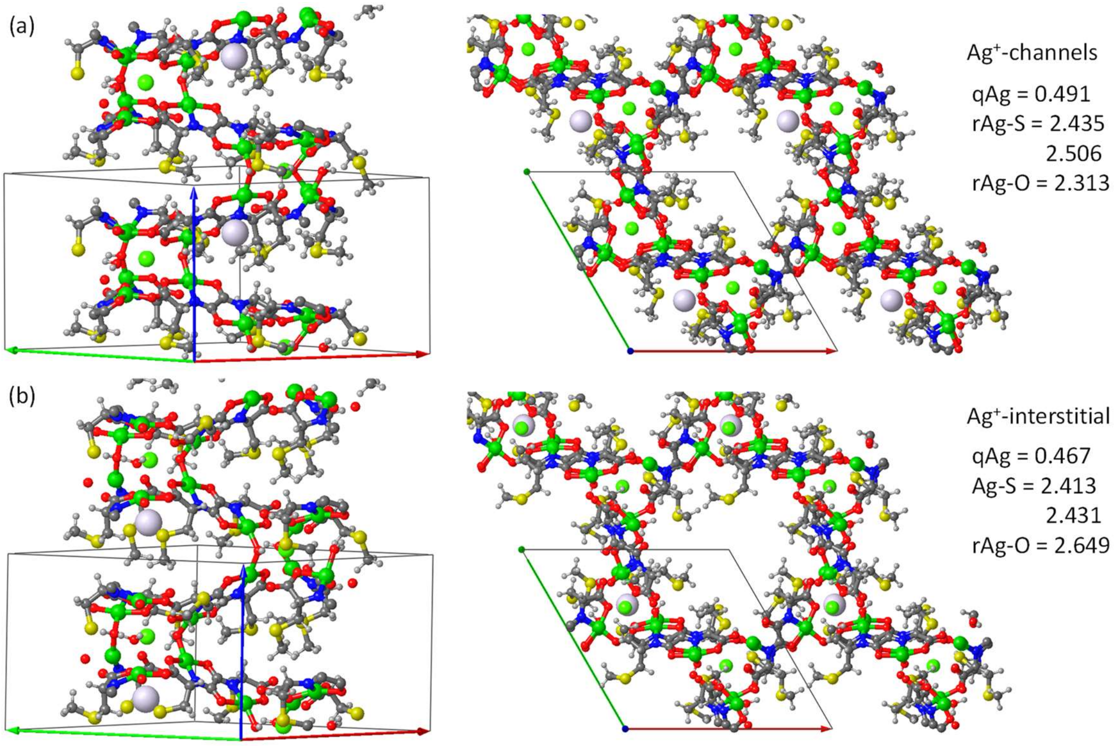

Figure S17. Optimized structure for **Ag^{+}@1** by DFT calculations. Bader charges (q) and bond distances (r) for Ag atoms (grey balls) in both channel (a) or interstitial positions (bottom) are also indicated.

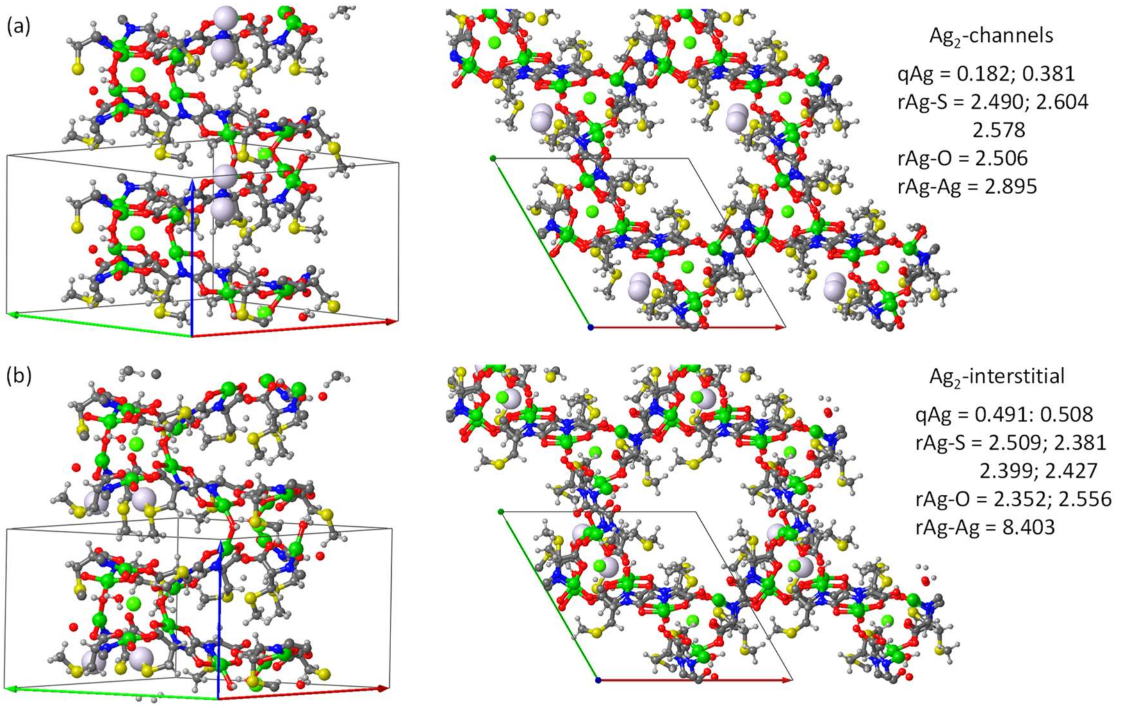

Figure S18. Optimized structure for **Ag⁰@1** by DFT calculations. Bader charges (q) and bond distances (r) for Ag atoms (grey balls) in both channel (a) or interstitial positions (bottom) are also indicated.

Supporting References

[S1] Mon M, Ferrando−Soria J, Grancha T, et al. Selective gold recovery and catalysis in a highly flexible methionine−decorated metal−organic framework. J. Am. Chem. Soc. 2016;138:7864−7867.

[S2] CrysAlisPro 1.171.38.41 (Rigaku Oxford Diffraction, 2015).

[S3] SAINT, version 6.45, Bruker Analytical X−ray Systems, Madison, WI, 2003.

[S4] Sheldrick GM. J. Appl.Cryst. 2015;48:3−10.

[S5] Sheldrick GM. Acta Cryst. 2015;C71:3−8.

[S6] Sheldrick GM. Acta Cryst. 2008;A64:112−122.

[S7] Farrugia LJ. J. Appl. Crystallogr. 1999;32:837.

[S8] Palmer D. CRYSTAL MAKER, Cambridge University Technical Services, C. No Title, 1996.

[S9] Kresse G, Furthmüller J. Phys. Rev. B 1996;54:11169.

[S10] Perdew JP, Burke K, Ernzerhof M. Phys. Rev. Lett. 1997;78:1396.

[S11] Blöchl PE. Phys. Rev. B 1994;50:17953.

[S12] Sanville E, Kenny SD, Smith R, Henkelman GJ. Comput. Chem. 2007;28:899.
